# Supplementary material for: Bounds to electron spin qubit variability for scalable CMOS architectures
Source: Nat Commun. 2024 May 20;15:4299. doi: 10.1038/s41467-024-48557-x (PMC11106088; doi:10.1038/s41467-024-48557-x)
Supplement: Supplementary file 1 — Supplementary Information [file 41467_2024_48557_MOESM1_ESM.pdf]

Supplementary Information

Bounds to electron spin qubit variability for scalable CMOS architectures

Contents

1. Supplementary Fig. 1
2. Supplementary Table 1
3. Supplementary Table 2
4. Supplementary Fig. 2
5. Supplementary Fig. 3
6. Supplementary Fig. 4
7. Supplementary Fig. 5
8. Supplementary Fig. 6
9. Supplementary Fig. 7

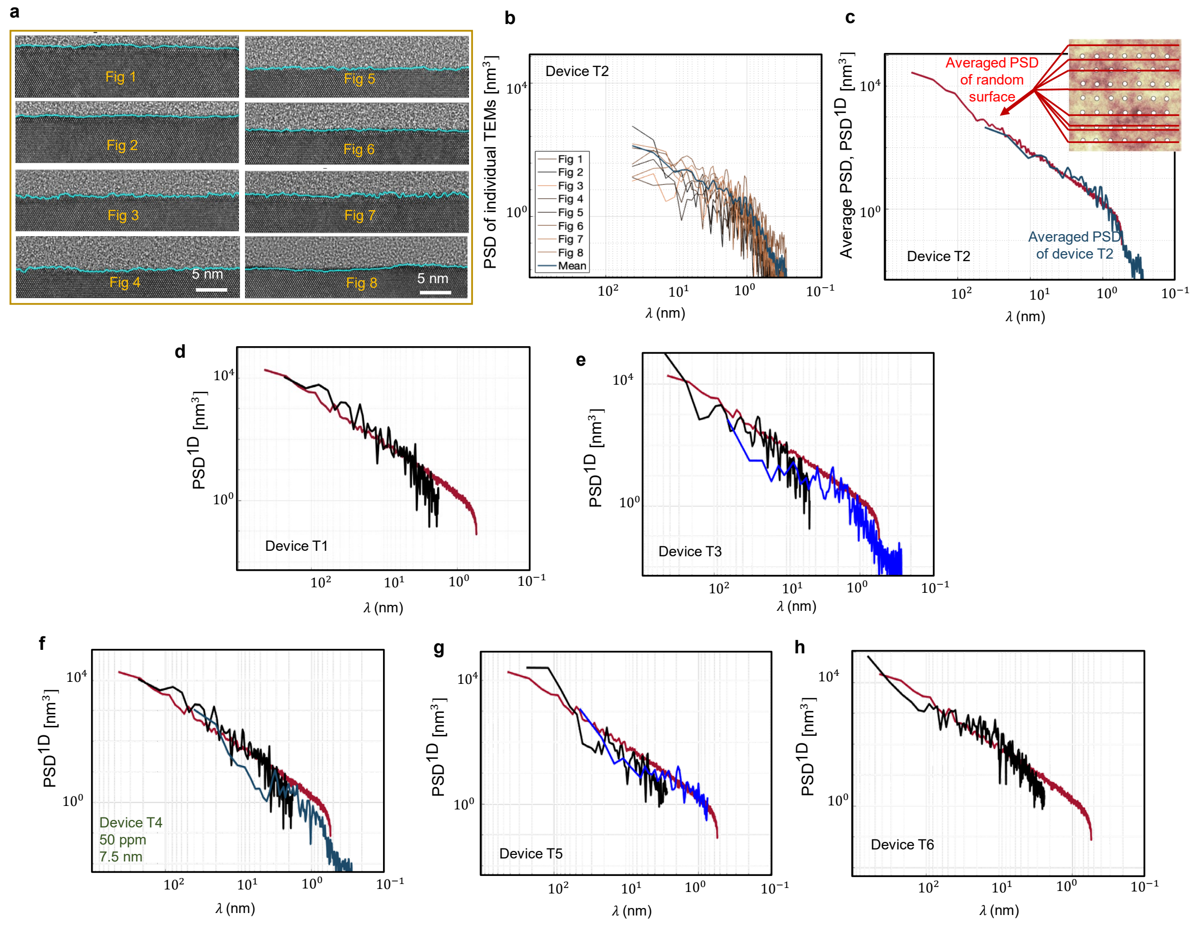

Supplementary Fig. 1: | **Characterization of oxide roughness in 6 devices.** **a**, 8 TEM images taken from device T2 with range ($\sim$ 40 nm) and interface fit. **b**, Individual PSD $\mathcal{C}^{1D}(\lambda)$ of each one of the TEM images. The blue line corresponds to the average PSD. **c**, Comparison of the average PSD of device T2 with the random surface. **d-h**, Characterization of the average PSD for the five remaining devices: **d** T1, **e** T3, **f** T4, **g** T5, **h** T6. The oxide characteristics of each device and the number of TEMs used to obtain each PSD profile is described in Supplementary Table 1.

Device	Si^{29} Purity [ppm]	Oxide Thickness [nm]	TEM Range [nm]	# of TEMs
T1	800	8.0	400	2
T2	800	8.0	40	8
T3	800	8.0	500, 70	2, 3
T4	50	7.5	250, 40	2, 4
T5	800	8.0	250, 50	2, 4
T6	800	8.0	700	3

Supplementary Table 1: Table of devices where transmission electron microscopy images (TEM) were taken. The TEMs capture the Si/SiO2 interface, showing the interface roughness. All oxides were grown under the same conditions except for device T4 consisting of a 7.5 nm SiO2 layer grown on isotopically purified 50pmm ^{29}Si. The PSD was obtained from TEMs with varying numbers and ranges, with more TEMs yielding a higher degree of precision in the PSD and RMS estimate.

**Gates**	dx_1/dV [nm V^{-1}]	dE_{z1}/dV [eV $nm^{-1}V^{-1}$]	dx_2/dV [nm V^{-1}]	dE_{z2}/dV [eV $nm^{-1}V^{-1}$]
P1	-6.74	13.42	-2.95	-2.11
P2	4.95	-0.68	5.5	14.75
J1	6.88	0.46	-3.57	-0.22
P3	0.04	-0.02	0.13	0.06
J2	0.02	-0.01	0.13	0.06

Supplementary Table 2: | **Impact of gate action on each quantum dot.** As a result of the harmonic fitting in Fig. 2 , we obtained the dependence of the dot parameters on the action of each gate. These numbers are used to estimate tunabilities of qubit parameters from atomistic simulations (see Methods section).

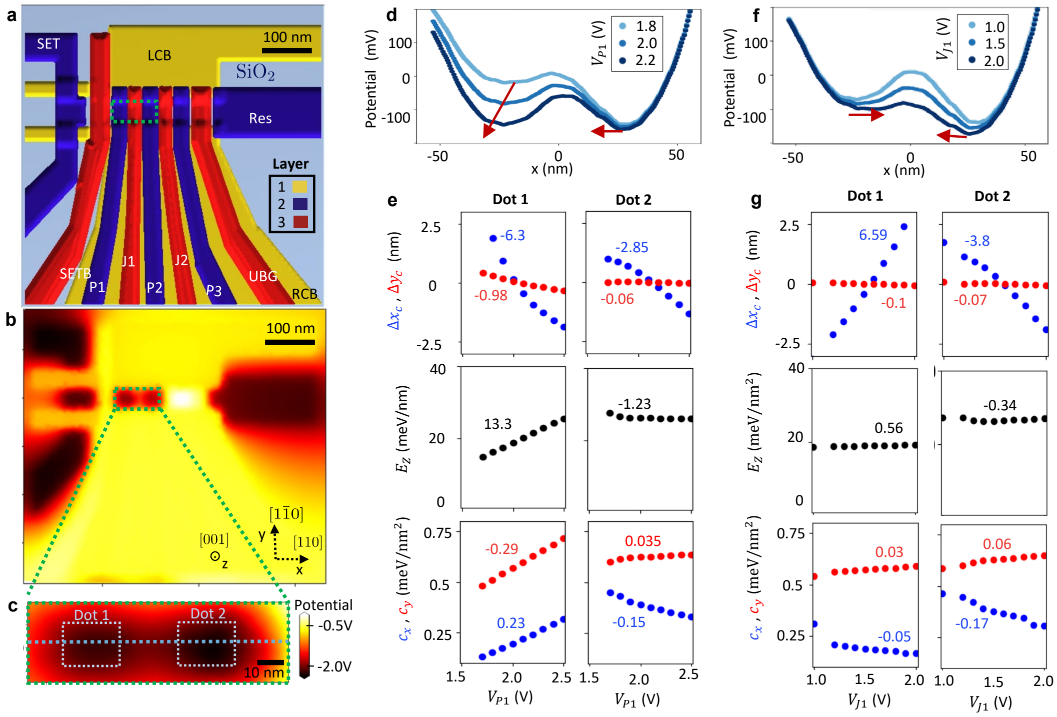

Supplementary Fig. 2: | **Potential simulations and gate-impact on quantum dots.** **a**, Horizontal view of the 3D device model that we input in COMSOL for potential simulations. The green square shows the region where the dots are formed. **b**, Potential landscape of the device simulated in COMSOL. The set of gate potentials is derived from experiments. A double quantum dot is isolated in the green square region bellow the gates $P1$ and $P2$.**c**, Zoom into the potential profile at the green region in **b**. Single dots are formed inside the white rectangles. We fit the potentials inside these regions to the harmonic model $V(x, y, z) = c_x(x - x_c)^2 + c_y(y - y_c)^2 + zE_z$. **d,f**, Evolution of the potential profile over the cyan line in **c** under the tuning of gates $P1$ (**d**) and $J1$ (**f**). **e,g**, Characterization of the impact of gates $P1$ (**e**) and $P2$ (**g**) on each quantum dot. We evaluate this impact over 5 variables: Displacement of the dot position from the mean $(\delta x_c, \delta y_c)$, transversal electric field E_z and curvatures (c_x, c_y). The numbers inside the plots show the slope of the curve. Their units depend on the variable evaluated, for instance , the unit of dx_c/dV is [nm/V].

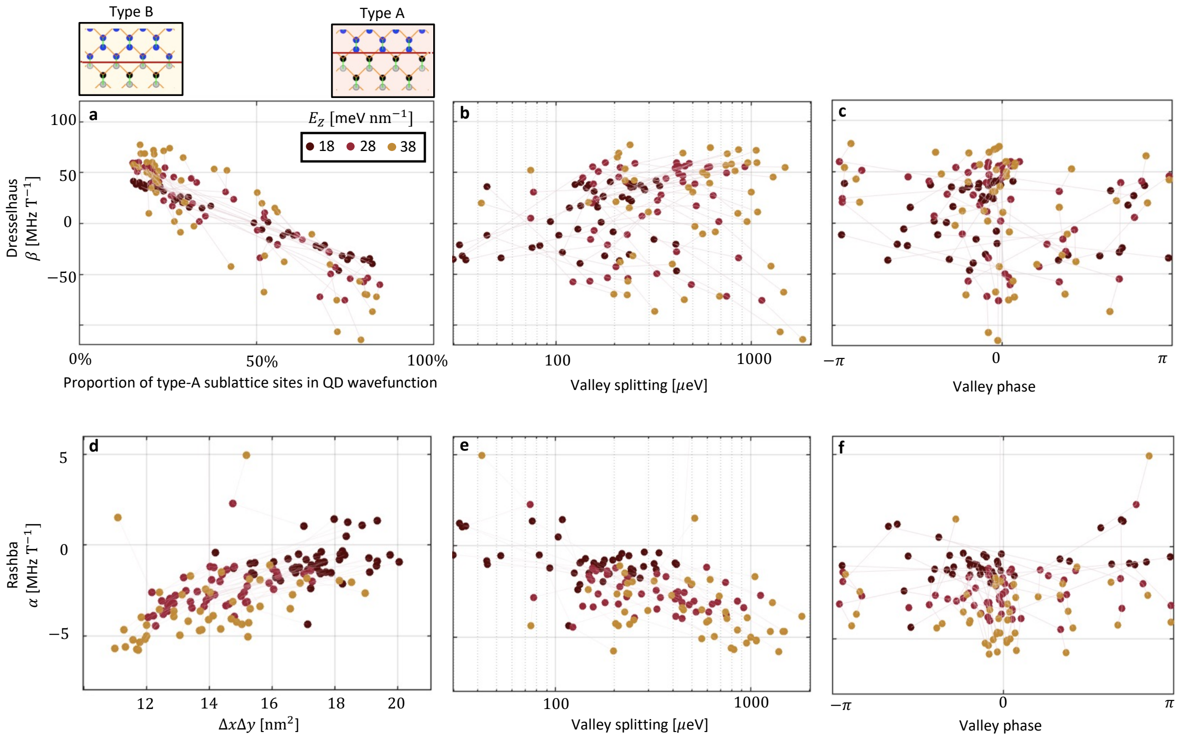

Supplementary Fig. 3: | **Investigating spin-orbit correlations.** **a-c**, Dependence of Dressselhaus term vs: **a**, Proportion of type A lattice sites on the quantum dot wavefunction. **b**, Valley splitting. **c**, Valley phase. **d-f**, Dependence of Rashba spin orbit coupling vs **d**, $\Delta x \Delta y$, where Δx (y) is the standard deviation of the $x(y)$ site in the ground state wavefunction $(\Delta x^2 = \langle x^2 \rangle - \langle x \rangle^2)$. **e**, Valley splitting. **f**, Valley phase. These plots do not include near degeneracy points as this data can disturb significantly the scale of the spin-orbit interactions. The leading correlations for each variable are plotted in the first column: Proportion of sub-lattice sites for Dresselhaus and quantum dot area for Rashba.

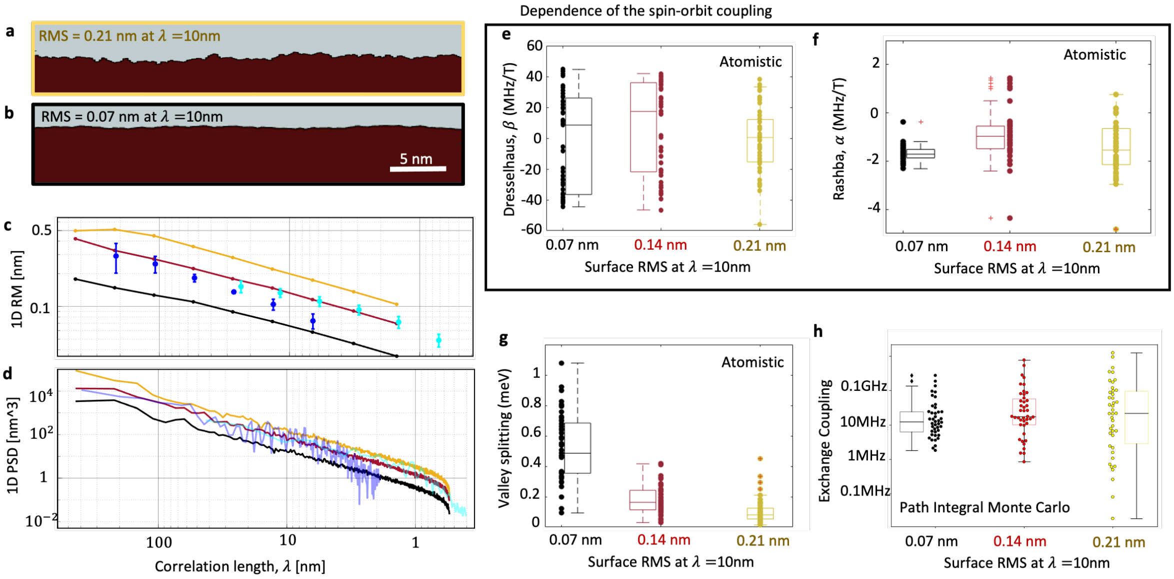

Supplementary Fig. 4: | **Dependence of qubit parameters on the surface RMS: a-b**, We generated two additional surfaces with higher (**a**) and lower (**b**) RMS that the one used in the paper to understand the potential benefits of improving the surface quality. **c-d**, 1D RMS and 1D PSD of the original surface and the new surfaces plotted in **a** and **b**. The profiles of devices T1 and T2 are also included in both figures for comparison with the dispersion of the measured data. **e-f**, Spin-orbit coupling dependence on the RMS. Dresselhaus values are slighly more dispersed for smoother interfaces as the results approach to the flat surface limits. **g-h**, RMS vs valley splitting(**g**) and two-dot exchange coupling (**h**). Smoother surfaces lead to higher mean valley splittings **g** and to smaller variability in exchange coupling **h**.

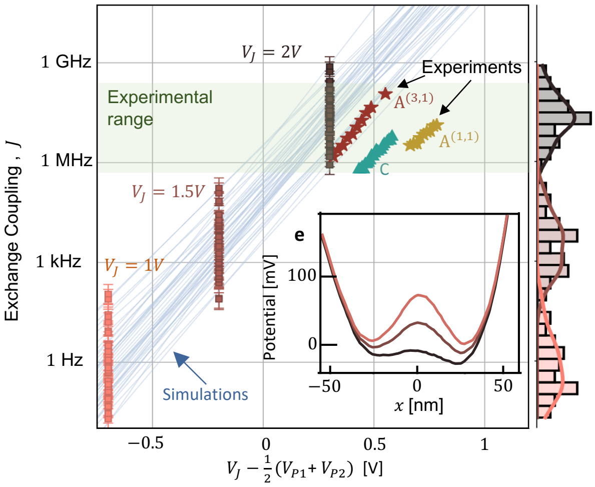

Supplementary Fig. 5: | **Exchange variability due to interface roughness (without charged traps)**. Interface roughness generates variability in exchange coupling of ∼3 orders of magnitude. The exchange baseline was slightly higher than in experiments. The inclusion of negative charge traps in the simulation led to a decay in the exchange baseline of 1.5 decades, giving a better agreement with experiments (Fig. 5 in the main text).

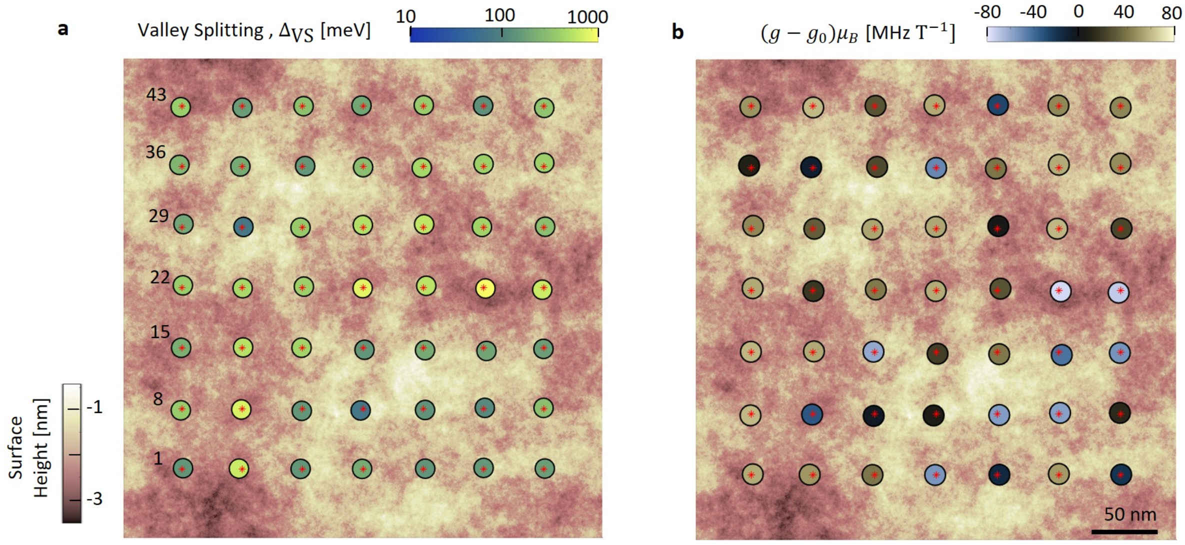

Supplementary Fig. 6: **Quantum dot parameters simulated with atomistic tight-binding of each dot in the 7×7 qubit array: a** Valley spliting (in logarithmic scale). **b** g-factor with magnetic field pointing to [110]. The simulated rough surface is plotted behind the dots for reference. Each dot was simulated with atomistic tight-binding in a simulation cell of 40 nm ×40 nm containing a section of the global rough surface. $E_z = 28$ meV nm^{-1} in these simulations.

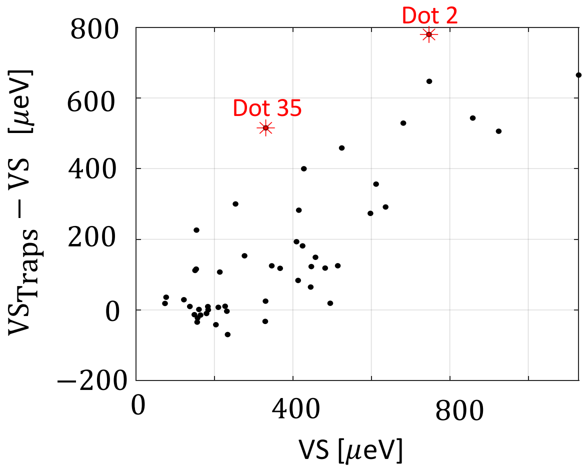

Supplementary Fig. 7: Correlation between shift of the valley splitting after the inclusion of charge traps ($\mathrm{VS_{Traps}} - \mathrm{VS}$) vs the valley splitting of the original dot VS. This indicates that in a dot with large VS, the valley splitting is potentially more susceptible to electric disorder. We highlight two data points that are out-of-range for typical values, which correspond to dots 2 and 35. As seen in the trap configuration in Figure 4 of the main text, both quantum dots have a negative trap in their vicinity close to the Si/SiO_2 interface.
